# Cytoskeleton-Dependent Transport as a Potential Target for Interfering with Post-transcriptional HuR mRNA Regulons

**DOI:** 10.3389/fphar.2016.00251

**Published:** 2016-08-17

**Authors:** Wolfgang Eberhardt, Amel Badawi, Abhiruchi Biyanee, Josef Pfeilschifter

**Affiliations:** Pharmazentrum Frankfurt/ZAFES, Klinikum der Johann Wolfgang Goethe-UniversitätFrankfurt am Main, Germany

**Keywords:** cytoskeleton inhibitors, HuR, RNA binding protein, mRNA stability, mRNA trafficking

## Abstract

The ubiquitous mRNA binding protein human antigen R (HuR), a member of the embryonal lethal abnormal vision protein family has a critical impact on the post-transcriptional control of AU-rich element bearing mRNA regulons implied in inflammation, senescence, and carcinogenesis. HuR in addition to mRNA stability can affect many other aspects of mRNA processing including splicing, polyadenylation, translation, modulation of miRNA repression, and intracellular mRNA trafficking. Since many of the identified HuR mRNA targets (“HuR mRNA regulons”) encode tumor-related proteins, HuR is not only discussed as an useful biomarker but also as promising therapeutic target for treatment of various human cancers. HuR which is most abundantly localized in the nucleus is translocated to the cytoplasm which is fundamental for most of the described HuR functions on target mRNAs. Accordingly, an elevation in cytoplasmic HuR was found in many tumors and correlated with a high grade of malignancy and a poor prognosis of patients. Therefore, direct interference with the intracellular trafficking of HuR offers an attractive approach to intervene with pathologically deregulated HuR functions. Data from several laboratories implicate that the integrity of the cytoskeleton is critical for HuR-mediated intracellular mRNA localization and translation. This review will particularly focus on drugs which have proven a direct inhibitory effect on HuR translocation. Based on the results from those studies, we will also discuss on the principle value of targeting cytoskeleton-dependent transport of HuR by natural or synthetic inhibitors as a potential therapeutic avenue for interfering with dysregulated post-transcriptional HuR mRNA regulons and related tumor cell functions. In spite of that, interfering with cytoplasmic HuR transport could highlight a so far underestimated action of microtubule inhibitors clinically used for cancer chemotherapy.

## Introduction

Modulation of mRNA turnover and translation reflect key paradigms of post-transcriptional gene regulation in physiology and pathology of higher eukaryotes. Besides mutations in the genome, tumor-related gene expression profiles frequently rely on alterations in post-transcriptional control mechanisms. Together with the non-coding RNAs, particularly the micro RNAs which either affect transcript stability or the level of the encoded protein, RNA binding proteins (RBPs) have a critical impact on various mRNA modalities (Keene and Tenenbaum, 2002; Keene, 2007). The ubiquitously expressed turnover and translation regulatory-RNA binding protein (TTR-RBP) human antigen R (HuR), a member of the embryonic lethal abnormal vision (ELAV) like protein family is one of the best characterized RBP in this scenario. In addition to mRNA stability and translation, HuR can affect additional RNA features namely splicing and intracellular transport of mRNA with the latter one being highly relevant for spatial control of protein synthesis (for review see: Tekotte and Davis, 2002; Martin and Ephrussi, 2009). Members of the Hu protein family, target many short-lived mRNAs preferentially those bearing destabilizing AU-rich elements (AREs) in their 3′or 5′untranslated regions (UTRs) which have also been summarized as post-transcriptional “Hu-mRNA regulons” (Ma et al., 1996; Myer et al., 1997; Pascale and Govoni, 2012). A large variety of these mRNAs encode proteins which implement hallmarks of cancer including proliferation, cell survival, immune evasion, angiogenesis, and metastasis (for review see: Mazan-Mamczarz et al., 2008; Abdelmohsen and Gorospe, 2010). Accordingly, an upregulation of HuR was observed in many tumors (López de Silanes et al., 2005; Abdelmohsen and Gorospe, 2010; Wang et al., 2013). It is for this reason that, HuR is considered a promising target of novel anticancer therapies. Nevertheless, keeping in mind that HuR is indispensable and necessary for normal life and development (Ghosh et al., 2009), approaches aiming at the total knockdown of HuR expression have to be considered with caution. Alternative strategies which would allow for only a temporal intervention with some specific HuR activies are therefore certainly much more desirable.

Similar to many other RBPs, HuR is a typical shuttling protein which translocates from the nucleus to the cytoplasm and backward (“HuR shuttling”). Pathologically, elevated cytoplasmic HuR levels in patients correlate with an increase in tumor size, higher invasion and poor outcome as convincingly demonstrated in ovarian (Erkinheimo et al., 2003), breast (Heinonen et al., 2005), and colorectal cancer, respectively (Denkert et al., 2006). Structurally, the bidirectional transport of HuR is related to the *H*uR *n*ucleocytoplasmic *s*huttling sequence (HNS) residing within the hinge region of the protein. Transport of HuR across the nuclear envelope requires an interaction of the HNS with components of the nucleo-cytoplasmic transport machinery, including transportins 1 and 2 (Rebane et al., 2004), importin-1α and the export route via the chromosome region maintenance 1 (CRM1) nuclear pore receptor also known as exportin 1 (Siddiqui and Borden, 2012). Importantly, in most cell types the shuttling of HuR is not a constitutive process but transiently induced by activation of different signaling devices (Kim et al., 2008; Doller et al., 2010). Accordingly, redistribution of HuR is a direct target of specific post-translational modifications including methylation (Li et al., 2002), and protein kinase dependent phosphorylation (Abdelmohsen et al., 2007; Doller et al., 2007, 2008). Importantly, these protein kinases, e.g., checkpoint kinase 2 (Chk2) and enzymes of the protein kinase C family have also been implicated in the regulation of tumor-related processes (for a review see: Doller et al., 2008).

## Cytoskleton Dependent Hur Transport

In addition to HuR’s nucleo-cytoplasmic transit, the specific cytoplasmic HuR localization, e.g., to the translation apparatus, the exosomes or the processing (P)-bodies for storage of mRNA is an important determinant for the destiny of the HuR-bound target mRNAs. Directed mRNA transport to a distinct cytoplasmic destination is furthermore highly relevant for spatial and temporal control of protein synthesis (Hovland et al., 1996; Tekotte and Davis, 2002; Martin and Ephrussi, 2009). Previous data demonstrated that the ubiquitously expressed HuR (Cheng et al., 2013; Doller et al., 2013) and its neuronal relative HuD (Fujiwara et al., 2011) can utilize either the actin- or microtubule-dependent cytoskeleton for transport of mRNA cargo. Interestingly, the nuclear export of HuR seems closely linked to its cytoplasmic trafficking since pharmacological disruption of either of both cytoskeletal transport systems prevents accumulation of HuR protein in the cytoplasm (Cheng et al., 2013; Doller et al., 2013). Targeting of both transport systems therefore represents a valuable approach to intervene with post-transcriptional ELAV/Hu mRNA regulons.

## Interfering With Microtubule-Dependent Hur Transport

A growing list of chemically diverse compounds perturbing the dynamics of microtubules have proven clinical efficacy in the treatment of various human cancers. The main therapeutic effect of currently used microtubule inhibitors seems to arise from cell cycle arrest due to inhibition of the mitotic spindle typically followed by an induction of apoptosis due to mitotic catastrophe (Jordan et al., 1996; Huszar et al., 2009). Microtubule destabilizing drugs as the vinca alkaloids vinblastine and vincristine from the Madagascar periwinkle *Catharanthus roseus* or, colchicine, an alkaloid derived from the autumn crocus *Colchicum autumnale* prevent the polymerization of microtubules (Huszar et al., 2009). In contrast, microtubule stabilizers such as paclitaxel or the hemisynthetic taxol docetaxel prevent the depolymerization of tubulin (Jordan and Wilson, 1998). Despite of their antagonistic effects on tubulin polymerization, all of these compounds are potent inhibitors of mitosis (Jordan and Wilson, 1998). In addition to affecting mitosis, microtubules are important for cell motility and for transport of organelles, vesicles and are relevant for long-distance transport of protein and mRNA (for review see: Holt and Bullock, 2009; Blower, 2013). The microtubule-RNA contact needed for RNA transport is mediated by formation of a ternary complex between the RBP, a microtubule-associated protein (MAP) and the zipcode which in most cases resides in the 3′UTR of the mRNA (Antic and Keene, 1998). Importantly, transport of an mRNA does not exclusively occur via one distinct cytoskeletal route but can switch between actin-and microtubule-directed transport system as demonstrated for β-actin mRNA (Martin and Ephrussi, 2009). A prominent example for microtubule-dependent mRNA transport is HIF-1α. Searching for novel pharmacological inhibitors for HIF1α, the administration of the indoline sulfonamide MPT0B098 a novel small-molecule inhibitor of microtubule polymerization to lung adenocarcinoma A549 cells efficiently impaired HIF1α-triggered gene expression (Cheng et al., 2013). These sulfonamide-based compounds were synthesized in order to overcome various modes of resistance and to achieve an improved pharmacological profile when compared to clinically established microtubule inhibitors (Nien et al., 2010). Mechanistically, MPT0B098 binds directly to the colchicine-binding moiety of tubulin. The destabilization of HIF1α mRNA by MPT0B098 was shown to be mainly due to inhibition of nucleo-cytoplasmic HuR shuttling (Cheng et al., 2013). Since HIF1α itself targets a large variety of genes, MPT0B098 interferes with the post-transcriptional expression of many tumor-related genes involved in tumor growth, cell metabolism, cell survival, and other functions. Furthermore, MPT0B098 has antiangiogenic properties as it affects the growth and density of microvessels in tumor specimens (Cheng et al., 2013). The pharmacological benefit of MPT0B098 is furthermore highlighted by strong antiproliferative effects even on multidrug-resistant human cancer cells (Nien et al., 2010). Similar to other microtubule-targeting drugs, MPT0B098 can induce cell growth arrest by avoiding the transition of cells from G2 to the M phase (Cheng et al., 2013). In contrast to MPT0B098, the microtubule stabilizing taxene docetaxel was found to increase HuR-mediated stabilization of COX-2 mRNA in the mammary epithelial cell line 184B5/HER mainly through and activation of the p38 mitogen activated (MAP) kinase and PKC mediated signaling mainly due to the induction of stress (Subbaramaiah et al., 2000, 2003). Notably, the stimulatory effect on COX-2 mRNA stability by taxanes demonstrated in this cell line is mainly relying on an increased HuR binding to the 3′UTR of the COX-2 mRNA. Since HuR phosphorylation by p38 MAPK and PKC can activate HuR-dependent stabilization on target mRNA (Lafarga et al., 2009; Doller et al., 2010) the net effect on HuR mRNA regulons by taxanes may critically depend on the extent of drug-induced activation of these stress kinases in a given cell type. In another study, microtubule perturbation by taxol or vinblastine inhibit HIF1α expression through the polysomal release of HIF1α mRNA although the authors did not formally demonstrate a direct correlation of the disrupted HIF1α mRNA transport with HuR (Carbonaro et al., 2011). Instead, a functional role of the microtubule-dependent cytoskeleton in the control of protein translation by HuR was highlighted by a report from Antic and Keene (1998). These authors demonstrated a critical role of microtubule-associated ELAV proteins in targeting mRNP particles from RNPs to the translation apparatus. Mechanistically, the interaction between ELAV proteins and microtubules is thought to be indirect and mediated, e.g., by MAPs. In case of HuD, the light chain of MAP1B (LC1) is linking the RBP-bound mRNA to microtubules, a process which is critical for the long-distance transport of ARE-containing mRNAs in neurons (Fujiwara et al., 2011). Given the plethora of tumor related genes targeted by HuR, it is plausible that in addition to interfering with tumor cell mitosis, an inhibition of post-transcriptional HuR regulons may essentially contribute to the potent antitumorigenic activity of clinically established microtubule inhibitors. In addition to the clinically established microtubule targeting drugs, microtubule-associated motor proteins, namely the kinesins have gained increased attention as novel targets for cytoskeleton-interfering therapies (Huszar et al., 2009). To date, more than 14 different kinesin protein families with an overall of 600 kinesins have been identified. Many of them are not only involved in mitosis regulation, but are highly relevant for microtubule dependent intracellular transport as well. Currently, there is pharmacological effort to test synthetic kinesin inhibitors in phase I and phase II trials in order to overcome side effects and drug resistance often observed with conventional microtubule inhibitors. It would be interesting to investigate some of the most promising kinesin inhibitors for their potential suppressive effect on HuR shuttling and related RNA trafficking.

## Interfering With Actomyosin-Dependent Transport

The actin-based cytoskeleton is involved in different cellular processes including cell shapening, cell motility, cell adherence, endocytosis and exocytosis, and intracellular transport of cargo. Therefore, it represents another attractive target for cancer chemotherapy (Jordan and Wilson, 1998). However, due to the lack of specificity for different types of actin, the use of most actin-targeting inhibitors tested so far have proven detrimental “off target” effects (Fenteany and Zhu, 2003). Nevertheless, compounds which preferentially act on cytoskeletal actin while having only week effects on cardiac, muscle, or smooth muscle actin represent attractive candidates for novel cancer therapy.

### Inhibitors of Actin-Polymerization

**FIGURE 1:**
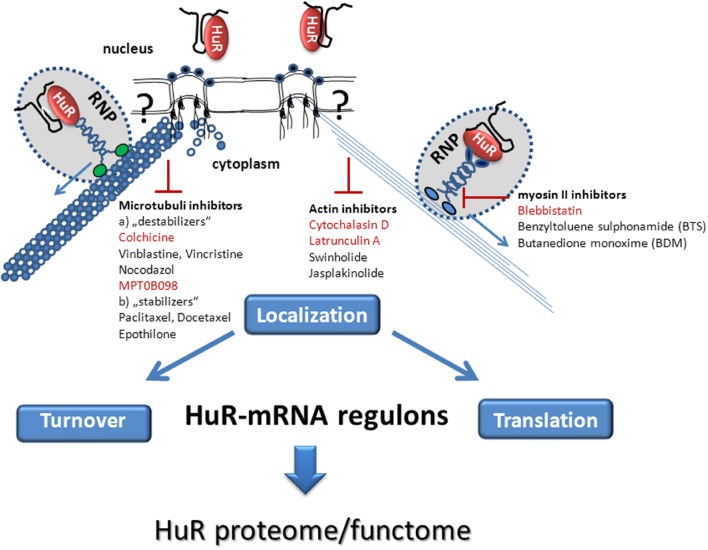
**Schematic summary of pharmacological stratagies aiming at the inhibition of post-transcriptional HuR mRNA regulons by interfering with cytoskeleton-dependent intracellular HuR-mRNA transport.** The cytoplasmic transport of HuR bound mRNA cargo occurs in form of a remodeled ribonucleoprotein (RNP) particle by a mechanism which requires either the actin-myosin based **(Right)** or microtubule dependent **(Left)** cytoskeleton. The hypothetical connection between cytoskeletal elements (microfilaments, microtubules) and the nuclear pore complex is indicated by a question mark. HuR bound target mRNAs are further directed to their final destination (turnover or translation) via a motor-driven transport along actin filaments **(Right)**, or along microtubuli **(Left)**. Pharmacological inhibitors of the actomyosin dependent cytoskeleton **(Right)** including actin inhibitors and myosin II inhibitors or drugs which affect the (de)polymerization of microtubules **(Left)** with a potential effect on cytoplasmic HuR transport are listed. Those cytoskeleton Inhibitors which have proven a direct effect on cytoplasmic HuR abundance in the literature are highlighted in red color.

Promising candidate compounds which may at least partially fulfill the above mentioned criteria are the marine derived macrolides latrunculin A and B isolated from the Red Sea sponge *Negombata magnifica*. Latrunculins reversibly bind to actin monomers by forming a stoichiometrically fixed 1:1 complex with G actin, thereby avoiding the assembly of actin monomers into filaments allosterically (Morton et al., 2000; Allingham et al., 2006). Due to a more definable mode of action, latrunculins are considered as more potent than the fungal cytochalasins which mainly act through binding to F-actin polymer thus preventing a further polymerization of actin monomers (Fenteany and Zhu, 2003). Pharmacologically, latrunculins by disrupting microfilament-mediated processes interfere with several tumor-related cell functions including angiogenesis, metastasis, cytokinesis, and proliferation. A strong anticancer effect *in vivo* has been demonstrated in a mouse xenograft model of human gastric cancer (Konishi et al., 2009). We previously have demonstrated a direct and critical involvement of actin-dependent HuR transport in the stabilization of some prototypical HuR mRNA targets in renal mesangial cells implicated in hypertension-induced fibrosis and inflammation (Doller et al., 2009, 2013). Importantly, the suppressive effects on cytoplasmic HuR accumulation and post-transcriptional HuR-mRNA regulons by latrunculin could be confirmed in hepatocellular carcinoma cells (Doller et al., 2015). The inhibition of cytoplasmic HuR by latrunculin presumably relies on the abrogated nuclear HuR export to the cytoplasm and implicates an actin-mediated communication between both compartments (**Figure 1**) which is also relevant for other RBPs (for a review see: Farina and Singer, 2002). In addition to the impaired HuR shuttling, latrunculin markedly decreased the stability of prototypical HuR targets including COX-2, cyclin A and cyclin D mRNAs concomitant with a significant reduction in the levels of corresponding proteins. In summary, we conclude that depending on which cell-type is analyzed, HuR utilizes either microfilament – or microtubule guided transport for targeting mRNA cargo to the translation apparatus. This raises the question of how binding to a specific mRNA may principally affect HuR’s association with preferentially one particular cytoskeletal tracking system. Data from our laboratory suggest that protein kinase-dependent phosphorylation of HuR confers a preferential HuR binding to only a subset of mRNA targets with a common zipcode in their respective 3′UTRs (Schulz et al., 2013). Similarly, these post-translational modifications may lead to a recruitment of HuR to a distinct motor (**Figure 1**). In addition to the latrunculins, a large arsenal of other actin inhibitors from marine organisms offers a large variety of bioactive compounds advantageous for novel anticancer approaches (Fenteany and Zhu, 2003). Those include the spongal cyclodepsipeptide jasplakinolide and the macrolides swinholide A, mycalodie B, and misakinolide A (**Figure 1**) which are comprehensively reviewed elsewhere (Fenteany and Zhu, 2003). Comparison of the inhibitory capacity of these actin inhibitors on intracellular HuR transport is an important issue which is currently addressed in our laboratory.

### Inhibitors of Non-muscle Myosin II

The myosin family of motor proteins represents an other attractive target for intervention with actomyosin-dependent cell motility and intracellular transport of cargo mRNA. Myosins have attracted significant attention and have come into the focus of pharmacological investigations for identification of putative targets of small molecule inhibitors. Members of the myosin superfamily are ATP-hydrolyzing motors mediating the contractility of actin filaments in muscle and non-muscle cells (for a review see: Sellers, 2000). Although the specific roles of non-muscle myosins is not fully understood they presumably play an important role in myosin-based cell motility (Fenteany and Zhu, 2003; Pertuy et al., 2014). In addition, myosin IIA (MyoIIA) is highly relevant for cell adhesion and migration (for a review see: Vicente-Manzanares et al., 2009). Since MyoIIA is also implicated in invasiveness of tumors it became a potential target of novel anti-cancer therapeutics (Duxbury et al., 2004). The majority of currently designed myosin inhibitors are non-competitive inhibitors of myosin ATPase activity which bind to myosin at an allosteric site outside of the ATPase binding pocket (Bond et al., 2013). Among those, the phenyl-pyrrolidinone derivative blebbistatin represents a highly selective inhibitor of non-muscle myosin II ATPase activity with almost no effects on the ATPase activity of other myosin motors (Allingham et al., 2005; Bond et al., 2013). In addition to a strong selectivity toward non-muscle myosin II, another important prerequisite for its principal use *in vivo* is its high cell permeability. Pharmacologically, blebbistatin by inducing an actomyosin complex disassembly impedes inflammatory infiltration which is an important feature of, e.g., progressive renal diseases (Si et al., 2010). Accordingly, blebbistatin can impair the invasion of pancreatic adenocarcinoma cells by interfering with the cell adhesion to the extracellular matrix (Duxbury et al., 2004). In line with these findings, we could previously demonstrate that in HepG2 cells, blebbistatin potently interferes with several HuR controlled cell functions including migration, invasion and the increased synthesis of prostaglandin E_2_ (Doller et al., 2015). Previously, we could identify the heavy chain 9 of non-muscle myosin IIA (MYH9) as a direct binding partner of HuR (Doller et al., 2013). The functional importance of MYH9 in the nucleo-cytoplasmic HuR shuttling and HuR related mRNA functions was confirmed by RNAi-mediated loss of function approach which showed the same inhibitory action on HuR activites as the pharmacological approach with blebbistatin (Doller et al., 2013, 2015). Similar to latrunculin, blebbistatin impaired the physical interaction of HuR with MYH9s indicating that ATPase activity is required for the interaction of myosin with HuR (Doller et al., 2015).

In a contrast to blebbistatin, the small myosin inhibitors *N*-benzyl-*p*-toluene *s*ulphonamide (BTS) and Butanedione monoxime (BDM) have a stronger inhibitory effect on skeletal muscle myosin II. However, the clinical use of these compounds is limited by the high concentrations needed to achieve myosin inhibition *in vivo* (Bond et al., 2013). Nevertheless, these compounds account as important lead compounds for the development of novel drugs for the treatment of diseases based on the overactivity or dysfunction of skeletal muscle myosin (Bond et al., 2013). Similar as with MPTOB098 and latrunculins, a more detailed pharmacological information including data about animal safety, pharmacokinetics and drug efficacy is also needed for these inhibitors for a better assessment of their potential benefit in the clinic.

## Conclusion

The list of genes regulated by the multifunctional RBP HuR is steadily growing and accordingly, its critical impact in carcinogenesis was validated for almost every type of human tumor. Therefore, HuR is considered a promising therapeutic target for treatment of human malignant diseases. Since the constitutively increased transport to the cytoplasm constitutes a major principle of post-transcriptional mRNA regulons by HuR, interfering with its subcellular redistribution reflects a valuable approach for targeting tumorrelevant HuR-mRNA regulons. Although cytoskeleton-dependent transport of Hu proteins was documented only by a couple of publications, our knowledge from directed mRNA transport implicates a stimulus- and cargo-specific transport of both Hu proteins along different cytoskeletal tracking systems critical for most of their cytoplasmic functions. In addition, current pharmacological antitumor concepts aiming at the inhibition of cytoskeletal dynamics should be reconsidered since some of the beneficial antitumor activities of these drugs may derive from inhibition of HuR. Besides the well-established microtubule inhibitors, the actomyosin dependent cytoskeleton represents another valuable target for anti-HuR strategies. The question whether HuR trafficking in tumor cells may generally depend on both types of transport systems, or is rather cell-type specific is an intriguing issue which warrants future investigation. Together with the inexhaustible source of natural actin inhibitors a large number of synthetic inhibitors of myosin and kinesin motors is principally available for targeting post-transcriptional mRNA regulons by HuR and related RBPs.

## Author Contributions

WE wrote the review, but all coauthors made substantial and intellectual contribution to the work as they had a major impact on the overall concept and furthermore approved it for publication.

## Conflict of Interest Statement

The authors declare that the research was conducted in the absence of any commercial or financial relationships that could be construed as a potential conflict of interest.
